# Genome-Wide Characterization and Expression Analysis of the *SBP-Box* Gene Family in Sweet Orange (*Citrus sinensis*)

**DOI:** 10.3390/ijms22168918

**Published:** 2021-08-18

**Authors:** Na Song, Yulin Cheng, Weiye Peng, ErPing Peng, Zengling Zhao, Tiantian Liu, Tuyong Yi, Liangying Dai, Bing Wang, Yanyun Hong

**Affiliations:** 1 College of Plant Protection, Hunan Agricultural University, Changsha 410128, China; songna@hunau.edu.cn (N.S.); dagyeah@yeah.net (W.P.); 18373140648@163.com (E.P.); zzling1999@163.com (Z.Z.); ltt20210719@163.com (T.L.); yituyong@hunau.net (T.Y.); daily@hunau.net (L.D.); 2 School of Life Sciences, Chongqing University, Chongqing 401331, China; yulincheng@cqu.edu.cn; 3 Hunan Provincial Key Laboratory for Biology and Control of Plant Diseases and Insect Pests, Hunan Agricultural University, Changsha 410128, China

**Keywords:** *Citrus sinensis*, *SBP* gene, genome-wide characterization, expression profile, abiotic and biotic stress

## Abstract

SBP-box is an important plant-specific transcription factor family and is involved in diverse biological processes. Here, we identified a total of 15 *SBP-BOX* genes in the important fruit crop sweet orange (*Citrus sinensis*) and characterized their gene structures, conserved domain and motif, chromosomal location, and *cis*-acting regulatory elements. *SBP* genes were classified into four subfamilies based on the amino acid sequence homology, and the classification is equally strongly supported by the gene and protein structures. Our analysis revealed that segmental duplication events were the main driving force in the evolution of *CsSBP* genes, and gene pairs might undergo extensive purifying selection. Further synteny analysis of the SBP members among sweet orange and other plant species provides valuable information for clarifying the CsSBP family evolutionary relationship. According to publicly available RNA-seq data and qRT-PCR analysis from various sweet orange tissues, *CsSBP* genes may be expressed in different tissues and developmental stages. Gene expression analysis showed variable expression profiles of *CsSBP* genes under various abiotic stresses, such as high and low-temperature, salt, and wound treatments, demonstrating the potential role of SBP members in sweet orange response to abiotic stress. Noticeably, all *CsSBP* genes were also downregulated in sweet orange upon the infection of an important fungal pathogen *Diaporthe citri*. Our results provide valuable information for exploring the role of *SBP-Box* in sweet orange.

## 1. Introduction

Sweet orange (*Citrus sinensis*) is one of the most popular and nutritive fruits in which the total global production of oranges can be up to 78.7 million tons (Food and Agriculture Organization of the United Nations, 2019). Citrus plants have acquired exquisite adaptive strategies to cope with geomorphological, climatic, and other cultivation factors. However, various biotic and abiotic stresses still caused high loss rates. Among them, melanose disease, caused by the *Diaporthe citri*, is a severe fungus disease and can infect all citrus cultivars, accompanied by degrading tastiness and appearance [1]. With the continuous progress of modern biotechnology, especially in recent decades, molecular breeding technology has gradually matured. Molecular breeding technology has the advantages of high efficiency, short period, and trait directional improvement [2]. Mining disease resistance and stress tolerance-related transcription factor gene have received increasing attention.

Accurate control of gene expression is vital to various biological processes. Of these mechanisms, gene transcription plays a key role. Transcriptional regulation is controlled by transcription factors (TFs), which are conventionally defined as sequence-specific DNA-binding proteins that control gene expression via activating or repressing downstream target genes at the level of transcription [3]. SQUAMOSA promoter-binding-protein (SBP) gene family is exclusively found in plants, and the members of this family all share a highly conserved DNA-binding domain, which is composed of approximately 80 amino acid residues and contains C3H or C2HC zinc-finger structure [4]. Conserved domain structural analysis of SBP revealed that a highly conserved nuclear localization signal partially overlapped with the zinc-finger structure at the C-terminal region [5]. *SPB* genes are known to be associated with the transcriptional regulation of various physiological processes related to growth and development and can regulate multiple plant adaptational responses to biotic and abiotic stresses [6]. In Arabidopsis, the *SPB* gene family showed remarkable functional diversity. *AtSPL8* affected fertility through control sporangia development [7], *AtSPL3/4/5* controlled timing of shoot developmental phenotypes and maturation [8], *AtSPL9* was found associated with both leaf growth rate and final size [9], the SOC1-SPL module involved in integrating hormone and photoperiod signals [10], and *AtSPL2/10/11/13* participated in the regulation of vegetative-reproductive transition [11]. In addition to these diverse functions in dicot plants, *SBP* genes also play a major role in various physiological and biochemical processes in the monocot plant. In rice, *OsSPL10* was preferentially expressed in the inflorescence stem and positively controlled trichome formation and salt tolerance [12], *OsSPL14* played a dual role in yield and immunity [13], and *OsSPL2/17/18* regulated fertile tiller numbers and plant height [14].

Accumulating evidence reveals the importance of *SBP* genes in stress tolerance and disease resistance [15,16]. MsSPL9 diminishes drought stress tolerance, at least in part, via regulating anthocyanin accumulation in *Medicago sativa* [17]. Silencing of *CaSBP08* resulted in enhancing the activity of *Capsicum annuum* defense enzymes as well the expression levels of pathogenesis-related genes and increasing resistance to *Phytophthora capsica* [18]. *OsSPL7* and *Ideal Plant Architecture1* (*OsSPL14*), two target genes of miR-156, positively regulated disease resistance against bacterial blight but negatively regulated yield [19]. Unfortunately, despite continuous efforts to infer the functions of development and stress response, the biological role and precise molecular mechanisms of the *SBP*-box gene family remains elusive. However, a comprehensive annotation of sweet orange *SPB* genes is the initial step toward fully elaborating underlying molecular mechanisms.

In this study, the genome-wide analysis of the *SBP* gene family in sweet orange is presented, including SBP gene family that is systematically identified, conserved structure, phylogeny, chromosomal localization, evolutionary history, and expression pattern in various tissues and under abiotic and biotic stress. Our studies lay the groundwork for the development for the abundant function of *SBP* genes in transcription regulation of sweet orange.

## 2. Materials and Methods

### 2.1. Isolation and Phylogenetic Analysis of CsSBP Genes in Sweet Orange

Sweet orange genome sequences were obtained from Joint Genome Institute (https://phytozome.jgi.doe.gov) (*Citrus sinensis* v1.1) and *C*. *sinensis* Genome Annotation Project (http://citrus.hzau.edu.cn/orange/index.php) (version2). Then, potential SBP candidates were isolated via homologous alignment among rice and Arabidopsis SBP family [20]. All resulting proteins were confirmed to contain SBP DNA-binding domain (pfam number: PF03110) using the Pfam database (Version 34.0, European Bioinformatics Institute).

SBP protein sequences of Arabidopsis, rice, and sweet orange species were aligned using ClustalW algorithm of MEGA-X software (version 10.2, Mega Limited, Auckland, New Zealand). To classify and illustrate the evolutionary relationship of SBPs identified from sweet orange, rice, and Arabidopsis, the phylogenetic analysis based on amino acid sequence alignments was conducted with MEGA-X software package using the neighbor-joining (NJ) algorithm and the bootstrap test was performed for 1000 re-samplings.

### 2.2. Chromosomal Location and Duplication Events among CsSBP Genes

All 15 *CsSBP* genes were located on the corresponding chromosomes of sweet orange using chromosomal locations information extracted from *C. sinensis* Genome Annotation Project with Toolkit for Biologists software [21]. Gene densities were drawn according to the genome annotation information. Multiple Collinearity Scan Toolkit X (MCScanX) was adopted to exhibit potential gene duplication events. The synteny relationship of the orthologous *SBP* genes between sweet orange, rice, and Arabidopsis were identified using Synteny visualization plugin embedded in TBtools.

### 2.3. Analysis of CsSBP Properties and Conserved Motifs

Information about physicochemical properties of the CsSBP protein, molecular weight, isoelectric point, and GRAVY was predicted using the DNAMAN and ExPASy-ProtParam proteomics server (http://web.expasy.org/protparam/) (Swiss Institute for Bioinformatics (SIB), Lausanne, Switzerland). The prediction of CsSBP protein tertiary structure analysis was performed using Protein Homology Recognition Engine V 2.0 (http://www.sbg.bio.ic.ac.uk/phyre2/html/page.cgi?id=index) (Structural Bioinformatics Group, Imperial College, London). The subcellular localization for CsSBP protein was predicted by Plant-mPLoc v2.0, an online software tool (http://www.csbio.sjtu.edu.cn/bioinf/plant-multi/). The conserved motifs were identified using Motif Elicitation (MEME, version 5.3.3, https://meme-suite.org/meme/tools/meme, National Institutes of Health) motif search tool. Conserved domain for SBP proteins from sweet orange was extracted using Batch CD-Search (https://www.ncbi.nlm.nih.gov/Structure/cdd/wrpsb.cgi) with default settings. The 2000 bp genomic DNA sequences upstream from each *CsSBP* genes transcription start site were chosen and submitted to PlantCare program (http://bioinformatics.psb.ugent.be/webtools/plantcare/html/), a portal to tools for the in *silico* analysis, to identify the *cis*-acting regulatory elements (CREs).

### 2.4. Plant Materials and Stress Treatments

The sweet orange plants collected from Hunan Agricultural University Planting Base were used in the stress treatment. Three different tissues (stem, leaf, and flower) of sweet orange were separately collected at the flowering stage. Fruit was sampled after maturity.

Four different stress treatment experiments with specific steps were conducted as previously described [22,23]. Each experiment was repeated three times.

A. Low- and high-temperature treatments. The well-growing sweet orange seedlings that were exposed to light at 25 °C were transferred to constant temperature incubator at 4 and 40 °C for low- and high-temperature treatments. Samples were harvested at 0, 12, 24, and 48 h after treatment.

B. High salt stress. The well-growing citrus seedlings were placed into a liquid medium containing 100 mM/L NaCl under high salt stress. Samples were harvested at 0, 12, 24, and 48 h after treatment.

C. Wounding treatment. The leaves of the well-growth citrus were gently wounded with a needle, leaving the non-wounding plants as the control group. Samples were harvested at 0, 12, 24, and 48 h after treatment.

D. *D. citri* inoculation. *D. citri* was inoculated on the oat medium and placed upside down in an incubator at 25 °C for 7 days. Until the mycelia completely covered the oat medium surface, the mycelium was separated from the oat medium by filtration and rinsed twice with double distilled water. The conidial suspension concentration was adjusted to 1.0 × 10^6^ per milliliter using a hemocytometer under a light microscope, and directly used spore suspension for inoculation. The inoculated plants were placed into the 25 °C plant growth chamber with 80% humidity for 5 days. Samples were harvested at 0, 24, and 72 h post inoculation.

### 2.5. RNA Isolation and Quantitative Real-Time PCR

Total RNA was isolated using TRIzol reagent according to the manufacturer’s instructions. RNA quality was verified by 1% agarose gels and the NanoDrop 2000 (Thermo Scientific, Wilmington, DE, USA). For the first-strand of cDNA synthesis, 2 μg RNA was reverse-transcribed in a reaction of 20 μL using the HiScript III All-in-one RT SuperMix Perfect for qPCR Kit (Vazyme, Nanjing, China). Each quantitative real-time PCR reaction was performed in a 20 μL volume containing 10 μL qPCR mix, 4 μL template DNA, 0.5 μL of each primer (10 μM), and finally adding RNase-free water to 20 μL. Each set of quantitative real-time PCR experiments were conducted in three biological replicates. Sweet orange COX gene was used as the internal reference gene. The quantitative PCR reactions were performed with the following cycling profile: 95 °C for 30 s, followed by 40 cycles of 95 °C/10 s and 60 °C/30 s. Melting curve analysis was executed to verify the specificity for each primer pair. The relative gene expression values of *CsSBP* gene family were calculated using the 2^−ΔΔCt^ method.

## 3. Results and Discussion

### 3.1. Identification and Phylogenetic Analysis of Citrus SBP Proteins

To identify SBP encoding genes in sweet orange, we initially performed a Blast search via different databases at the whole-genome level. Further screening by the Pfam scan was conducted to ensure the presence of conserved SBP domain (PF03110). A total of 15 SBP genes and 27 transcription factor variants were identified after removing the redundant sequences and were named based on their chromosomal location (Table S1). The number of SBP genes was consistent with other dicotyledons, such as the castor bean [24], physic nut [25], pepper [26], and tomato [27], and was far less than monocotyledonous plants wheat (48) [28] and moso bamboo (32) [29], which could be a consequence of a whole-genome-wide duplication event after the divergence of monocot and dicot plants. Alternative splicing is an important feature of eukaryotic organisms, different transcripts of the same SBP genes are represented by a, b, and c in this study (e.g., CsSBP1a, CsSBP1b, CsSBP1c). Family member silico analysis showed both amino acid sequence lengths, and partial physicochemical properties varied considerably. Most of SBP members were predicted to localize in the nucleus, and a small part had a transmembrane helix and was predicted to localize in the cytoplasm. In addition, a three-dimensional (3D) protein structure was dominated by the random coil with a small part α-helix and overall conformation remained largely unchanged except for a few different transcripts (Figure S1). Protein physicochemical properties and 3D structure changes were probably accompanied by functional diversity.

**Figure 1:**
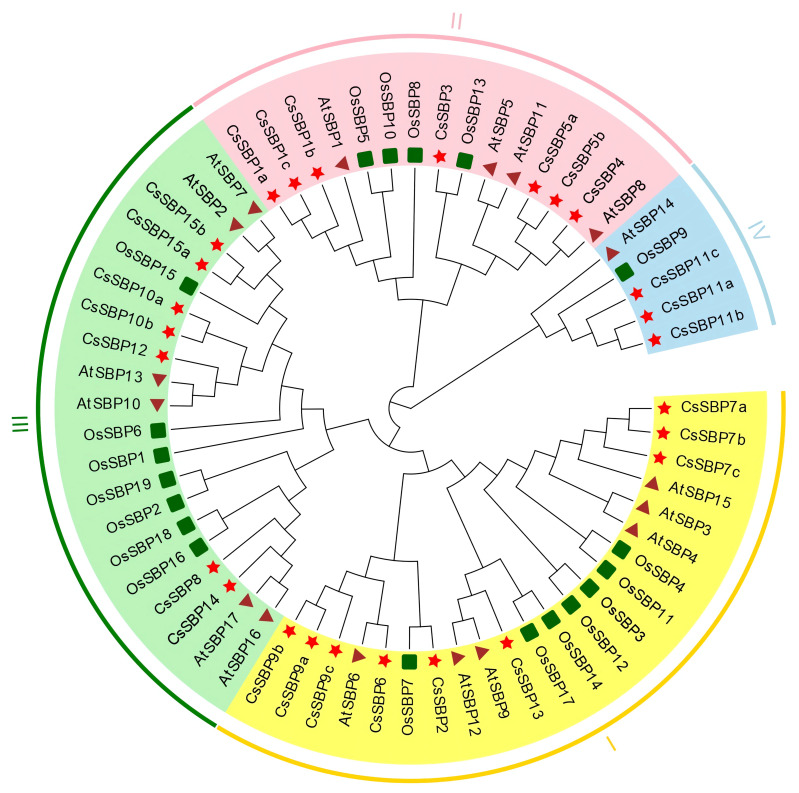
Phylogenetic tree of *Citrus sinensis*, *Arabidopsis thaliana*, and *Oryza sativa* SBP proteins. The tree was constructed from amino sequences using MEGA-X software by the neighbor-joining program with 1000 bootstrap replicates. Clades with different colors represent diverse subgroup.

In order to investigate the evolutionary relationships of the SBP family, a NJ phylogenetic tree was constructed of the aligned amino acid sequences from C. sinensis (15 SBP), Arabidopsis thaliana (17 SBP), and Oryza sativa (19 SBP; Table S2). As evident from the phylogenetic tree, the SBP family can be grouped into four subfamilies (I–IV; Figure 1). Subfamily I with seven members was the highest and subfamily II and III contained four and three members, respectively, whereas subfamily IV possessed only one member (CsSBP11), a similar grouping pattern occurred in Chinese jujube [30], suggesting independent evolution may have happened on CsSBP11.

### 3.2. Chromosomal Locations, Evolutionary Origin, and Divergence of CsSBP Genes

**Figure 2:**
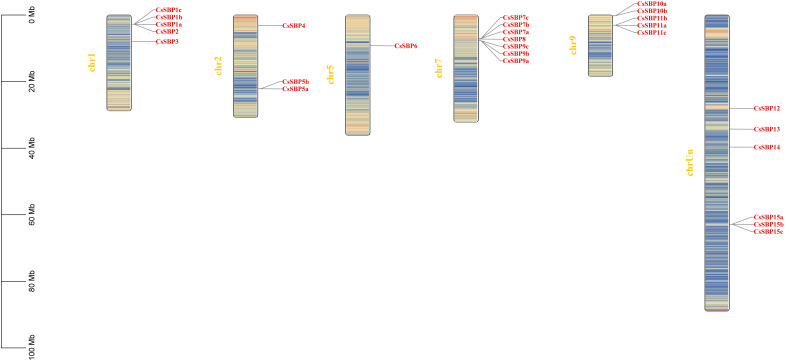
Chromosomal location of *CsSBP* genes. The scale represents 100 Mb chromosomal distance. The chromosome numbers are labeled above them. Gene densities were drawn based on the annotation information of *C. sinensis* genomes.

Chromosomal location with gene density analyses revealed that 15 CsSBP genes were unevenly distributed across six chromosomes (Chr 1, 2, 5, 7, 9, and Un; Figure 2). Uncharacterized Chromosome (Un) harbored the largest number of CsSBP genes (4), second greatest was Chr 1, Chr 7 (3), followed by Chr2, Chr 9 (2), whereas Chr 1 encompassed only one (CsSBP6). Other than that, the majority of CsSBP genes were located on relatively high gene density regions. 

**Figure 3:**
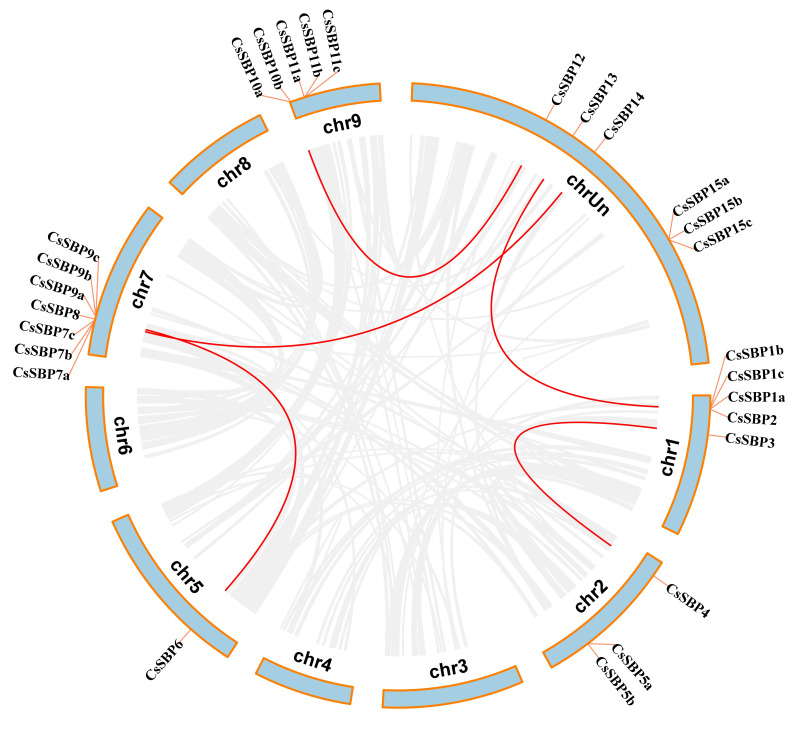
Duplication events of *CsSBP* genes. Circle plot showing duplication events of *CsSBP* genes on *C. sinensis* chromosomes. Red lines indicate duplication of *CsSBP* genes.

To characterize the expansion patterns of the CsSBP genes family, we surveyed the duplicated events within the sweet orange genome and comparison with chromosome position of all the CsSBP genes. Eventually, five duplication events with 10 CsSBP genes were identified in six chromosomes (Figure 3). We further calculated the Ks and Ka values of CsSBP gene pairs and found that all duplicated pairs ω (Ka/Ks) had a value < 1 (Table S3). In addition, divergence times of CsSBP gene pairs were between 6.42–11.03 million years ago (MYA). Taken together, these results indicated that duplication events provided the primary power for the evolution of sweet orange SBP genes family and these gene pairs under moderate purity selection.

**Figure 4:**
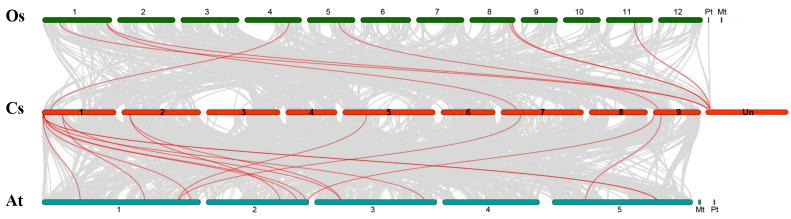
The homologous gene pairs between sweet orange and Arabidopsis and sweet orange and rice. Gray lines in the background indicate the collinear blocks within sweet orange and Arabidopsis and sweet orange and rice genomes. Red lines indicate homologous gene pairs.

We constructed a comparative syntenic analysis between sweet orange and Arabidopsis or rice genomes to obtain more insight into the evolutionary origin and divergence of the sweet orange SBP genes (Figure 4). According to this syntenic analysis, 7 and 15 collinear SBP gene pairs were identified in the rice and sweet orange and Arabidopsis and sweet orange pairs, respectively. The number of ortholog pairs of CsSBP-OsSBP was less than that of CsSBP-AtSBP, indicating that the divergence between rice and the common ancestor of dicotyledonous plants occurred before the division of citrus and Arabidopsis (Table S4). Apart from that, based on the phylogenetic tree and syntenic analysis, ortholog pairs *OsSBP9-CsSBP11-AtSBP14* were in the same evolutionary branch, suggesting these genes may impart similar function. The homologous gene *AtSBP14* has been reported to induce the expression of miR398 and regulate copper homeostasis [31].

### 3.3. Structural Analysis of SBP Family Genes in Sweet Orange

**Figure 5:**
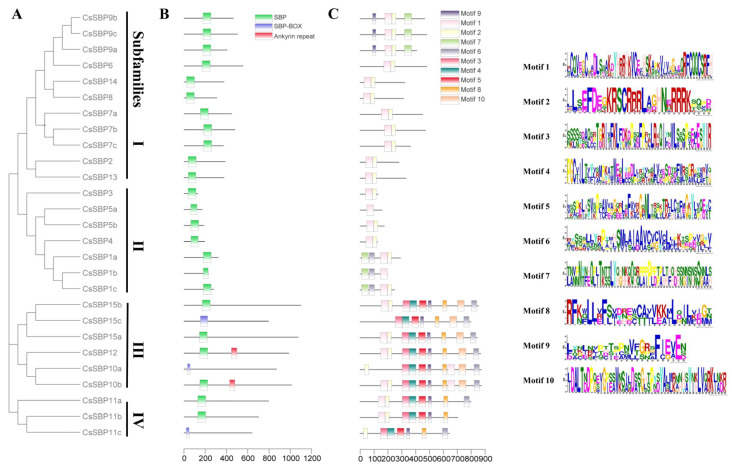
Phylogenetic relationships and protein structure of sweet orange CsSBP protein. (**A**) Neighbor joining trees constructed for CsSBP. (**B**) Conserved domains. Different domains are represented in different colors. (**C**) Protein motif. Schematic diagram of the conserved motifs of SBP proteins in sweet orange, which were elucidated using MEME. Each motif is represented by a number in the colored box. Logo of each motif is on the right side of the figure.

**Figure 6:**
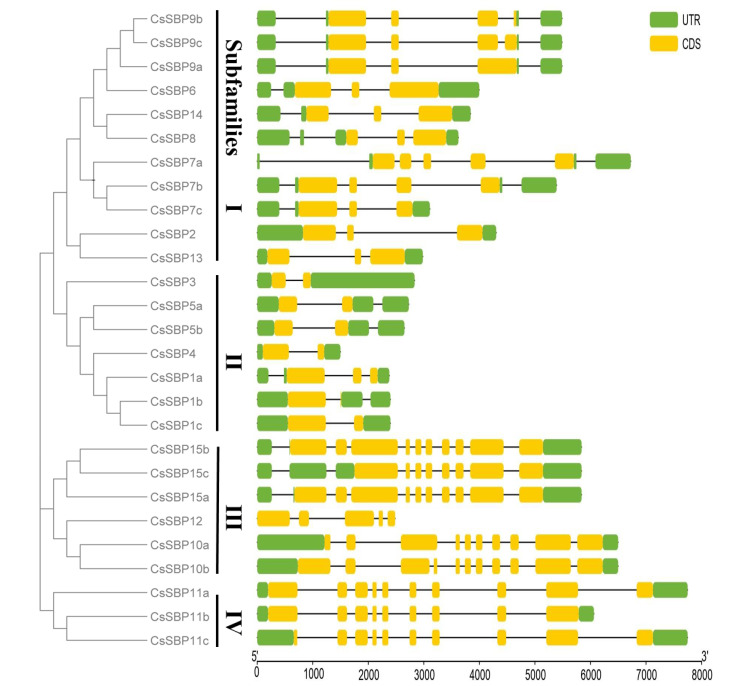
Gene structure of sweet orange *CsSBP* genes. Exons, introns, and untranslated region (UTR) are indicated by yellow rounded rectangles, black lines, and green rounded rectangles, respectively. The scale bar at the bottom is used to estimate the sizes of protein structure and gene structure.

Gene biological functions often link with their gene expression patterns and structure, and the gene structural differences were also significant determinants to support phylogenetic grouping of *SBP* gene family. The phylogenetic tree, constructed with *CsSBP* genes family, and the classification pattern was consistent with that in Figure 1 (Figure 5A). The complete SBP domain was present in all members except for the groups for certain variants (*CsSBP10a*, *CsSBP11c*, and *CsSBP15c*; Figure 5B). *CsSBP12* and *CsSBP10b* contained an additional complete ankyrin repeat domain, with low complexity sequence repeats, which is visualized by Dot-plots (Figure S2A). Ankyrin-repeat-domain-containing genes generally have diverse and complex biological functions because ankyrin repeats function to mediate protein-protein interactions [32]. In Arabidopsis, AtSPL14 possesses both SBP and Ankyrin repeat domain and has an effect on growth phenotype and in sensitivity to fumonisin B1 [33]. Ten motif logos were scanned, which is provided in the Figure 5C. Conserved motif distribution analysis revealed that subfamily III/IV motifs were the most enriched; by contrast, subfamily II members had short amino acid sequences and less motif. Certain motifs were group-specific: Motif 10 was only found in the subfamily III, suggesting that they may possess unique mechanisms and functions in sweet orange. Gene structure analysis revealed that *CsSBP* genes family varied greatly in gene structure, such as a maximum of 11 exons (*CsSBP10b*) and the fewest exons with only 1 (*CsSBP1b*; Figure 6). Gene structure of variants was more variable, not only in exon numbers but also in exon length. The amino acid sequence differences between the variants are displayed by dot-plots (Figure S2B). It is clear from the results that transcription elongation and termination altered the gene structure and domain gain and loss, thus affecting the functions of proteins.

### 3.4. Prediction of CRE-Involved Pathways, CsSBP Targets of miRNA

CREs in the promoter region play an essential role in response to stress and phytohormones, the 2 kb sequence upstream to promoter regions of CsSBP gene family was extracted for analysis (Figure S3). CREs of each CsSBP gene are provided in Table S5. Types of cis-elements are categorized into two groups: stress responses (low-temperature, drought, and defense) and phytohormone responses (auxin, methyl jasmonate, and salicylic acid). Analysis results indicated different CREs amounts and types among CsSBP gene family members and in different transcripts of the same SBP gene. Each CsSBP gene contained several types of CREs, particularly CsSBP11 and CsSBP15, which had significantly more repetitive elements (MeJA and SA responsive elements). As stress response hormones, SA is crucial for plant defense against pathogen infections and JA stimulates both plant stress response and elicitation of secondary metabolism [34]. Thus, CsSBP11 and CsSBP15 may be involved in disease resistance response.

A large amount of data indicate that miRNAs execute diverse functions by targeting SBP genes, especially miR156 family, which may be a major determinant of their fate [35]. In order to search possible targets of miRNA and target sites, we submitted CsSBP genes as candidate sequences that perform the RNA target analysis by the server standards. The prediction results showed that nine CsSBP genes were predicted targets of six miRNAs (Table S6), including the three mRNA/SBP gene populations (miR156, miR157, and miR529) that have been proposed multiple times, and three putative mRNAs (miR414, miR830, and miR5658) that have rarely been reported. miR5658 plays a key cross-point in the cucumber green mottle mosaic virus disease-resistance networks [36].

### 3.5. The Expression Patterns of CsSBP Genes in Various Tissues

**Figure 7:**
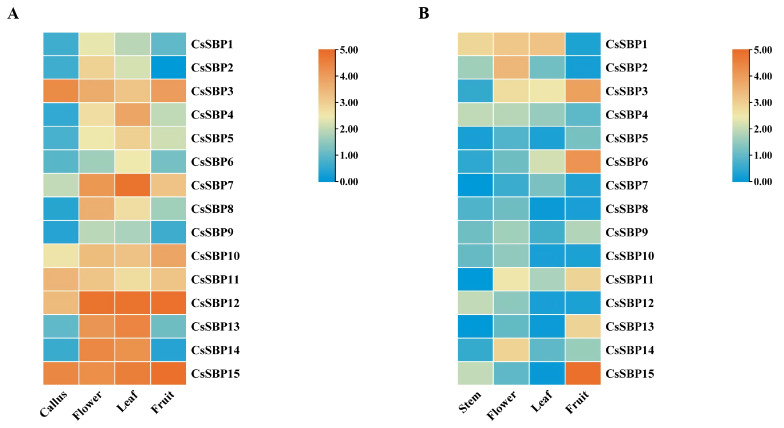
Heatmap showing the expression of *CsSBP* genes in different tissues. (**A**) Gene expression in the RNA-seq Data. (**B**) qRT-PCR analysis of *CsSBP* genes expression in different tissues. The log_2_ fold change values were used to generate heat maps. Color gradient from orange to blue indicates that expression values change from high to low.

The patterns of gene expression often have a correlation with its encoded protein function. Publicly available high-throughput sequencing data of four sweet orange tissues (callus, flower, leaf, and fruit) were used to assess the transcript abundance of the CsSBP genes [37]. According to the transcriptome sequencing data, CsSBP genes are ubiquitously expressed in different tissues (Figure 7A). Five CsSBP genes (CsSBP3, 10, 11, 12, and 15) shown have preferential expression in all four tissues, while the opposite was CsSBP9 at low levels across the four tissues examined. Overall, most of the CsSBP genes were highly expressed in flower and leaf but none was highly expressed in callus or fruit. Unfortunately, transcriptome sequencing data on the developmental stages of the sweet orange were not found. Since plant distinct developmental stages have different gene expression profiles, validation using quantitative real-time PCR (qRT-PCR) to investigate at the exact developmental stages (stem, flower, leaf, and fruit) are warranted [38]. While there were similarities between the transcriptome data and qRT-PCR results, there were also notable differences (Figure 7B). In terms of qRT-PCR, CsSBP genes had lower expression levels in flower and leaf. Through a comparative analysis and by using both assays, CsSBP2, CsSBP3 and CsSBP11 showed a similar expression pattern, while CsSBP7, CsSBP10, and CsSBP12 tissues transcript abundance quantified by qRT-PCR were exhibited much lower than that in transcriptome data. Furthermore, both transcriptome data and qRT-PCR results showed similarities in CsSBP12 gene expression of fruit tissues. Gene families evolved multiple times and they may have a loss or gain of function effect, or the same function but different spatial and temporal expression patterns [39]. Meanwhile, different members of CsSBP gene family may be expressed in various tissues, developmental stages, or environmental conditions, due to the noncoding regions with the high divergence level, despite significant sequence similarity of the coding regions and the encoded proteins with similar biological functions.

### 3.6. Expression Profiles of CsSBP Genes under Abiotic and Biotic Stresses

**Figure 8:**
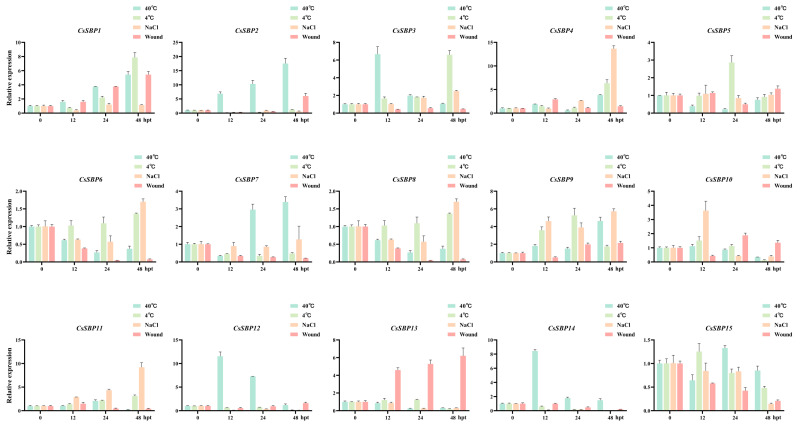
Expression levels of *CsSBP* genes under abiotic stress treatment. The Y-axis indicates the relative expression level; X-axis (0, 12, 24, and 48 hpt) indicates hours post abiotic stress treatment. Different colors represent different stress treatments. The standard errors are plotted using vertical lines. The experiments in all panels were repeated three times with similar results.

Diverse abiotic stresses, such as high salinity, wounding, and extreme temperature, may result in great losses of both production and marketability for sweet orange worldwide. The vast majority of plants that react to environmental variation rely mainly on the regulation of transcription factors affecting stress sensor response genes [40]. For example, miR156/SPL module regulates apple salt-stress early responses through activating the expression of MdWRKY100 [41]. Beyond this, the miR156-SPB network has also been shown to be involved in plant high- and low-temperature stress response [42,43]. Expression profiles allowed us to select sweet orange SBP genes that play important roles in plant abiotic and biotic stress responses. The expression patterns of CsSBP genes show that the same CsSBP gene can be induced or repressed while subjected to the different stresses (Figure 8). *CsSBP1, CsSBP4*, and *CsSBP9* exhibited relatively high transcript levels during all four abiotic stress conditions and thus may be proposed as candidate genes potentially involved in abiotic stress signaling and tolerance. In addition to this, *CsSBP2*, *3*, *7*, *12*, and *14* was upregulated under heat treatment, *CsSBP3*, *5*, and *11* for low temperature treatments, *CsSBP3*, *8*, *10*, and *11* for salt treatment, and *CsSBP2*, *10*, and *12* for wound treatments. In contrast, *CsSBP15* was downregulated at all time points under four abiotic stresses. Given the different expression pattern of the SBP family under various stress treatments, it is suggested to play multiple roles in sweet orange abiotic stresses responses. 

**Figure 9:**
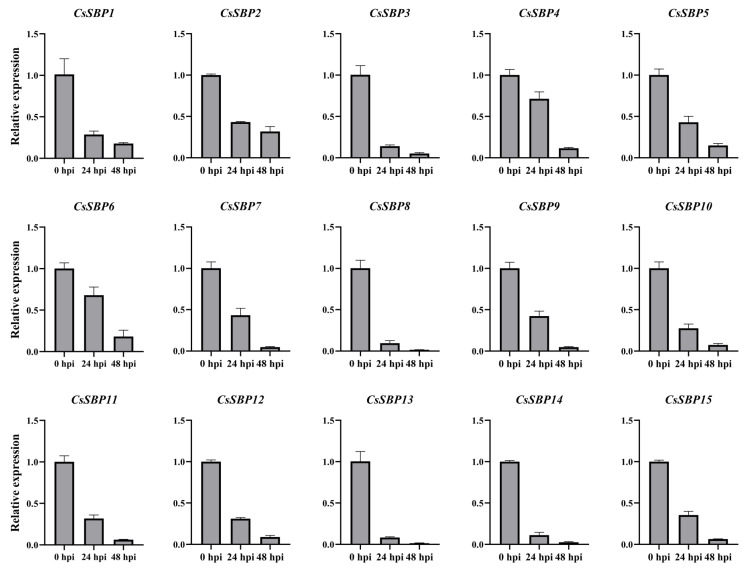
Expression levels of *CsSBP* genes inoculated with *Diaporthe citri*. The Y-axis indicates the relative expression level; X-axis (0, 24, and 48 hpi) indicated hours post *D. citri* inoculation. The standard errors are plotted using vertical lines. The experiments in all panels were repeated three times with similar results.

To examine the potential role of CsSBP genes in sweet orange defending against the pathogen D. citri, CsSBP genes changes in relative gene expression were validated by qRT-PCR. However, CsSBP genes had the same decreasing tendency of expression patterns, indicating that convergent evolution of disease-resistance function in the SBP family has occurred (Figure 9). The previous studies demonstrated that CaSBP gene family is an important negative regulator of immune responses to pepper. For the first time, it was reported that CaSBP12 increased pepper defense responses to Phytophthora capsic, and the tobacco plants overexpressing CaSBP12 showed a greater sensitivity to P. capsici infection with higher electrical conductivity and malondialdehyde accumulation compared with the wild-type [44]. Additionally, silencing of CaSBP11 enhanced the response against the P. capsica and induced the transcript level of defense-related genes, while in Arabidopsis, it may affect the expression of immune hormone genes that is involved in the SA or JA-mediated signaling pathways, to different degrees [45]. A similar situation occurred with Arabidopsis immune responses, AtSPL9 negatively regulated the accumulation of defense metabolites and had high sensitivity to Pseudomonas syringae [46,47].

## 4. Conclusions

In this study, we present genome-wide identification and characterization of CsSBP genes in the sweet orange genome. A total of 15 CsSBP genes phylogenetically divided into 4 subfamilies, which was further supported by the results of conserved domain and motif composition and gene structure. Moreover, analysis of CREs, miRNA target, protein tertiary structures, evolutionary origin, and divergence contributed to more in-depth inquiry. Expression profile analysis revealed that CsSBP genes may play diverse biological functional roles in multiple tissues. CsSBP genes not only had the characteristics of spatiotemporal specific expression but can also be induced or repressed by abiotic and biotic stress. The results presented here may contribute to future exploration of the biological functions of CsSBP genes, while also aiding in the selection of appropriate candidate genes that have functional roles in stress resistance.

## Data Availability

Data is contained within the article or Supplementary Materials.

## Supplementary Materials

The following are available online at https://www.mdpi.com/article/10.3390/ijms22168918/s1: Figure S1. Prediction of the tertiary structure of SBP protein. Confidence in the model above 95%. Figure S2. Dot plots of group analysis. (A) Repeat regions of ankyrin repeat domain. (B) Graph of dot plot shows the distinction between different transcripts. Figure S3. Cis-regulatory elements in the promoter of CsSBP genes. Different colors represent distinct putative elements. Table S1. In Silico analysis of CsSBP genes. Table S2. List of SBPs from Citrus sinensis, Oryza sativa, and Arabidopsis thaliana. Table S3. Tandem duplications and segmental of CsSBP gene pairs in Citrus sinensis. Table S4. Synteny analysis between Citrus sinensis and Arabidopsis thaliana and Oryza sativa. Table S5. The sequence and the predicted cis-elements of CsSBP promoter region. Table S6. miRNA targets of CsSBP genes. Table S7. qRT-PCR primers in this study.
