# Supplementary figures and images for: Genome-Wide Characterization and Expression Analysis of the SBP-Box Gene Family in Sweet Orange (Citrus sinensis)
Source: Int J Mol Sci. 2021 Aug 18;22(16):8918. doi: 10.3390/ijms22168918 (PMC8396319; doi:10.3390/ijms22168918)

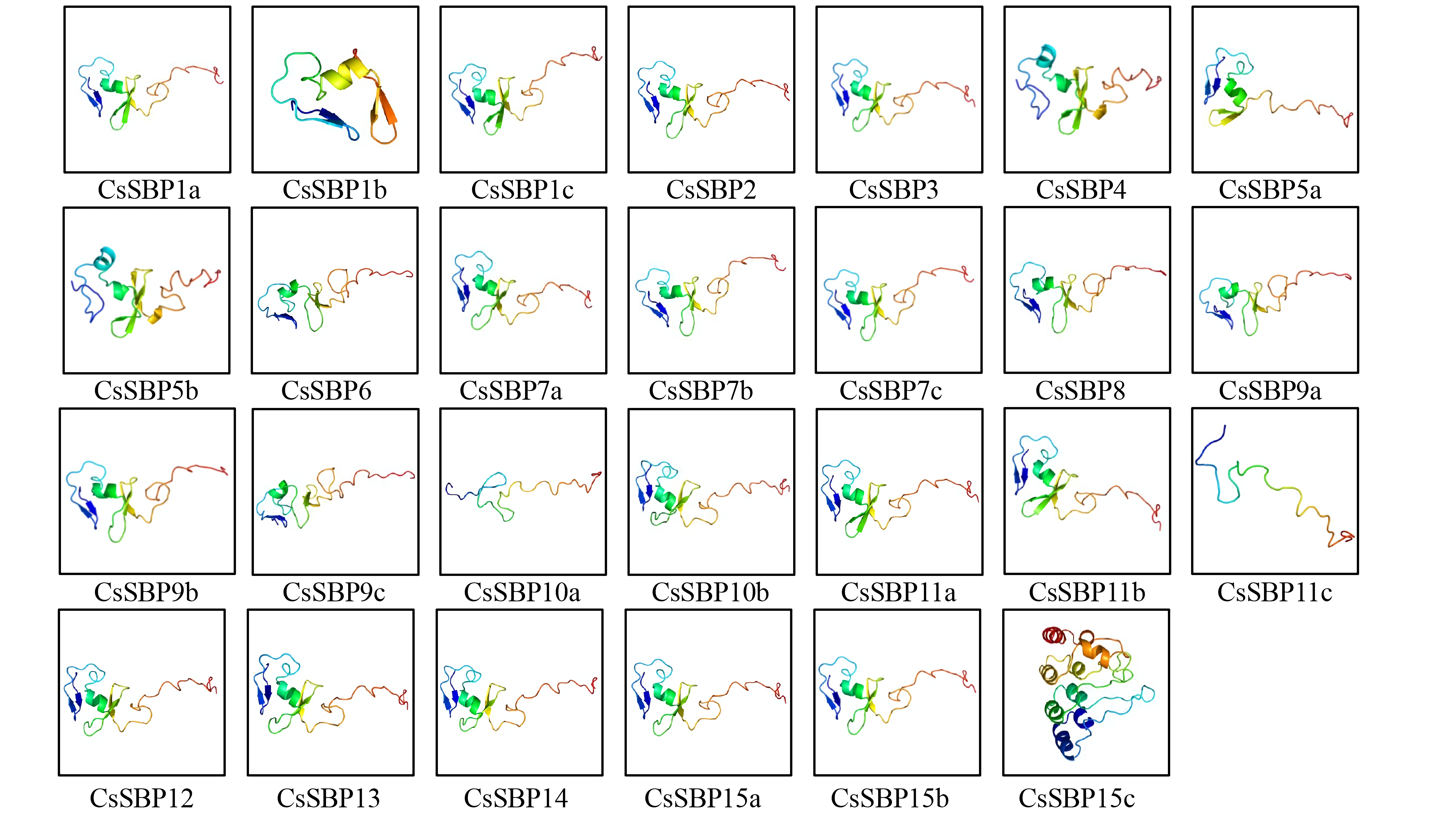

Supplement: Supplementary file 1 [file ijms-22-08918-s001.zip › Figure S1 SBP 3D(Confidence in the model above 95%).jpg]

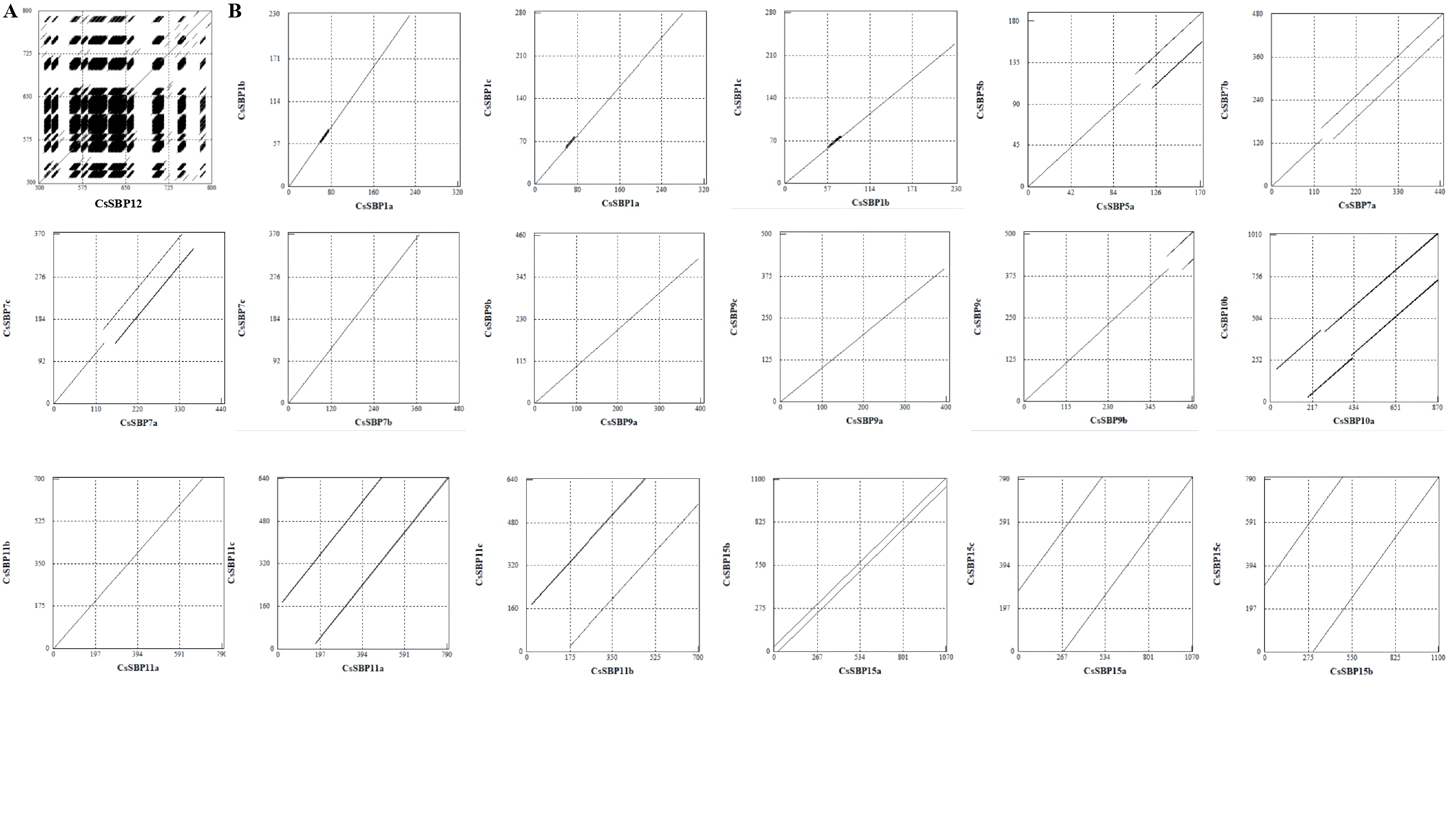

Supplement: Supplementary file 1 [file ijms-22-08918-s001.zip › Figure S2 Graph of dot plot shows the distinction between of different transcripts_page-0001 (1).jpg]

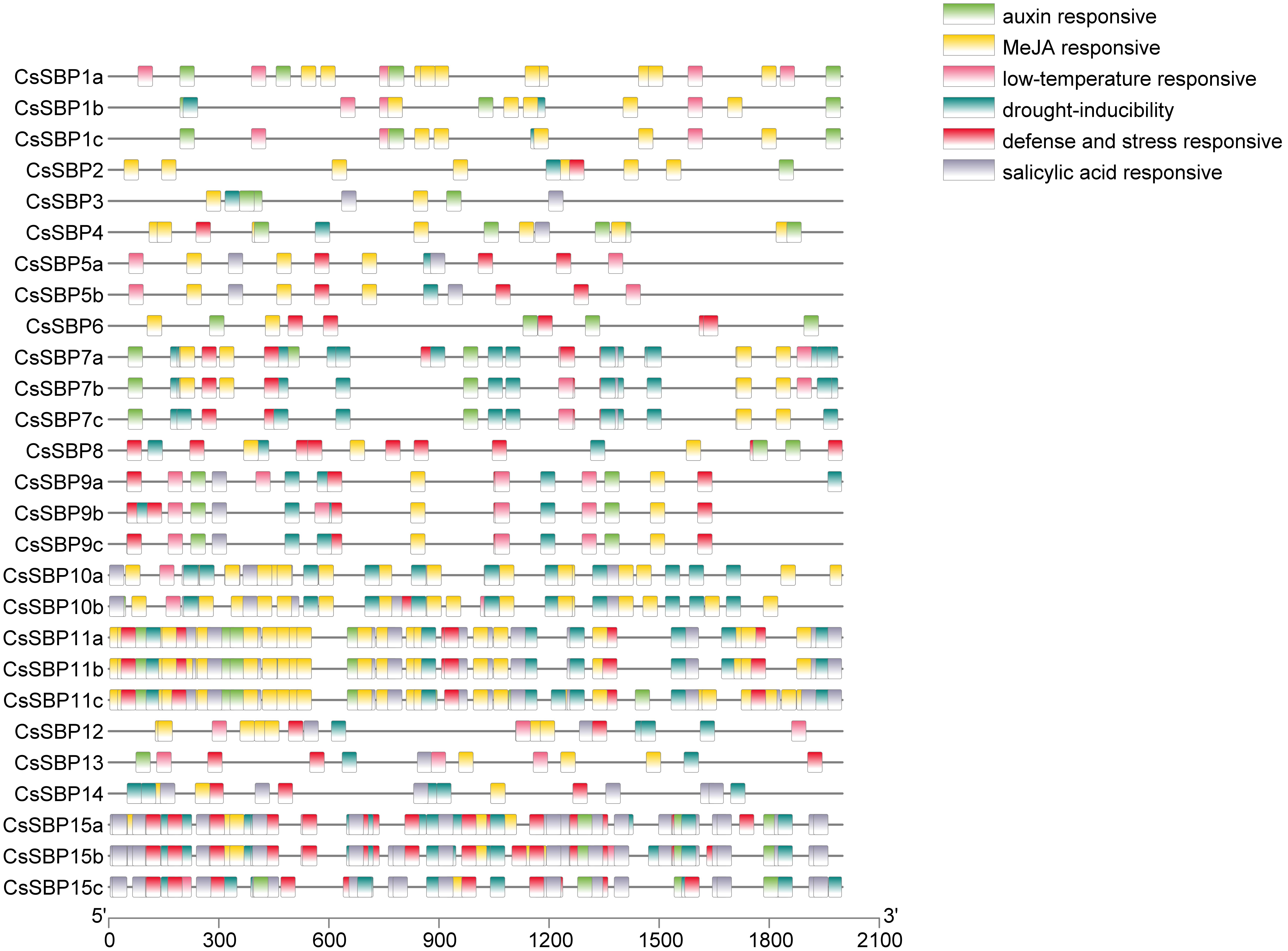

Supplement: Supplementary file 1 [file ijms-22-08918-s001.zip › Figure S3 Cis-regulatory elements in the promoter of CsSBP genes. Different colors represent distinct putative elements..jpg]
